# Supplementary material for: Development of a Machine Learning Model to Discriminate Mild Cognitive Impairment Subjects from Normal Controls in Community Screening
Source: Brain Sci. 2022 Aug 28;12(9):1149. doi: 10.3390/brainsci12091149 (PMC9497124; doi:10.3390/brainsci12091149)
Supplement: Supplementary file 1 [file brainsci-12-01149-s001.zip › brainsci-1836762-supplementary.pdf]

Supplementary Material

Data collection

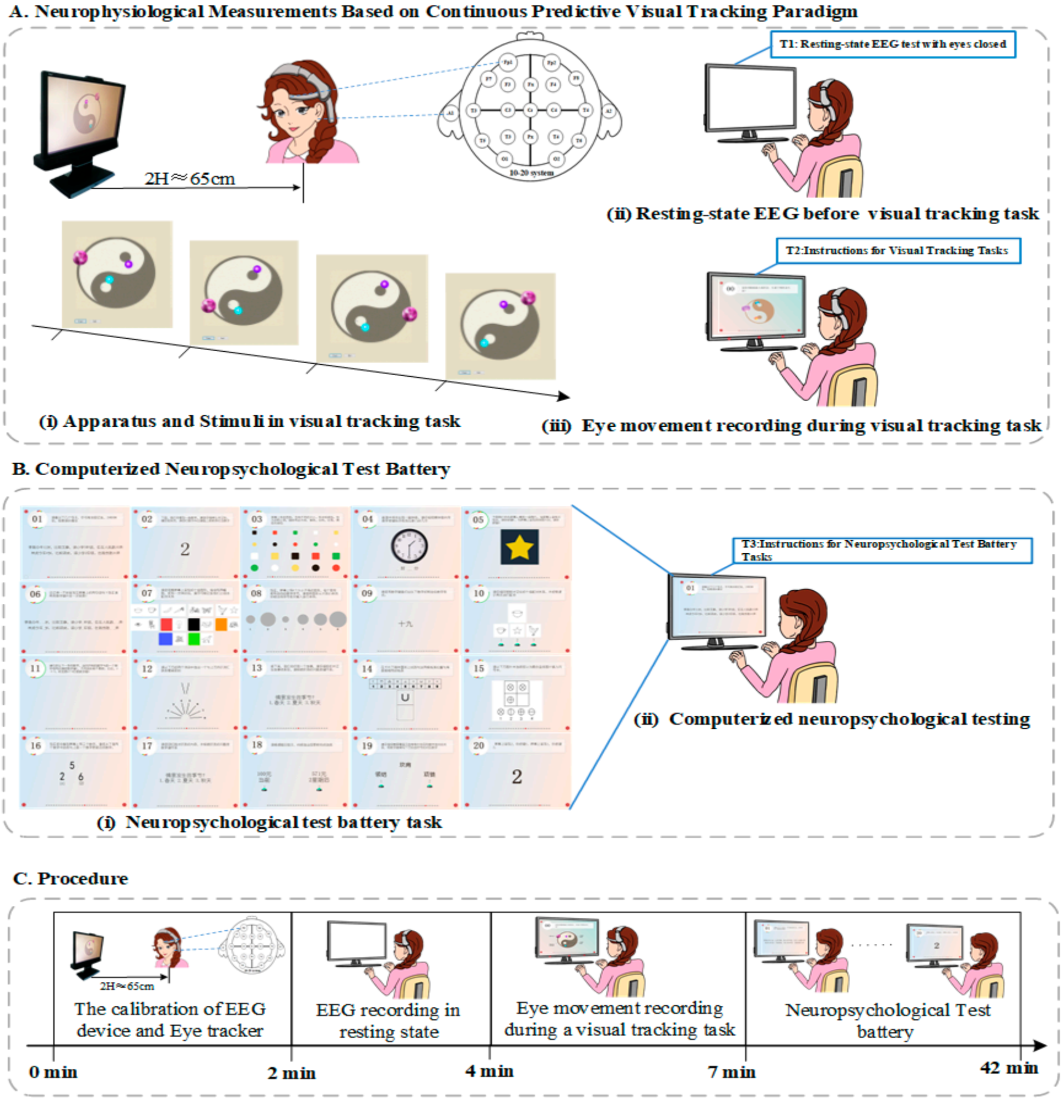

Figure S1. Data collection in this study. (A)Neurophysiological measurements Based on continuous predictive visual tracking paradigm; Visual stimulus, the stimulus consisting of three moving balls was presented on a personal computer with a 23′ monitor and viewing distance of approximately 65 cm from the monitor, in such a way that the distance between them and monitor was of 2H, H being the height of the displayed images. (B) Computerized neuropsychological test battery: Computerized neuropsychological tests were consisting of 20 tasks including memory, language, visuospatial, attention, executive function, and social cognition;(C) Procedure: Subjects were measured by EEG device and Eye tracker after the calibration of above devices. Before visual tracking task, the resting state EEG of 2 minutes were measured. Eye movement recording of 3 minutes were measured during visual tracking task. Computerized neuropsychological tests of 35 minutes were measured.

The schematic illustration of an experimental trial was shown in Figure A1. All subjects were arranged to sit on a comfortable chair in a room with diffuse light and noise-isolated wearable EEG device. Recordings during relaxed wakefulness were obtained from subjects in order to minimize muscular artifacts and with eyes closed for 2 min. The EEG device

mentioned above was single-channel Mind Wave MW001, which was produced by Neuro sky Inc., San Jose, CA. Two dry sensors were used to detect and filter the EEG signals of subjects. The sensor tip detected electrical signals from the forehead of the brain (corresponding to Fp1 in the 10-20 system) and the second sensor, ear clip, was a grounds and reference (corresponding to A1 in the 10-20 system), which allowed Think Gear chip to filter out the electrical noise.

After the initiation of the EEG device, a 9-point eye-tracking calibration step was started in which the subjects followed a moving target on the display screen. Following the calibration (approximately about 120 s), subjects were measured by EEG device and recorded their eye movements by Tobii EyeX eye tracker during the test phase. Eye tracker recorded gaze position in a monocular tracking mode providing 55 samples per second. Gaze position reports the (x, y) coordinates of a subject's gaze on the display (resolution: 1920 x 1080) in actual display coordinates (pixels) with origin (0, 0) at the top left.

The EEG was completed over 2 minutes while in the resting state and the ET data were then collected while performing the visual tracking tasks. Subjects were asked to follow the trace of purple ball consecutively regardless of the motion of the other two small balls and the Eye movement data during task execution were measured respectively. After visual tracking task, subjects were asked to follow instructions to complete 20 tasks of computerized neuropsychological test battery.

Computerized Neuropsychological Test Battery

Table S1 listed neuropsychological Test used in this study.

TABLE S1. Neuropsychological Test Battery in this study

Neuropsychological Task	Cognitive Domains	Task Time(s)	Record contents
Logical Memory Task	Memory	90	Accuracy, Time
Counting Backwards Task	Attention	44	Accuracy, Time
Visual Object and Space Perception Battery	Visual Perception	150	Accuracy, Time
Clock Reading Task	Visuospatial	65	Accuracy, Time
Set-shifting Task	Attention	140	Accuracy, Time
Short-term Task	Memory	20	Accuracy, Time
Visual Association Test	Visual Perception	180	Accuracy, Time
Wired Task	Executive Function	150	Accuracy, Time
Symbol Transformation Task	Semantic Comprehension	100	Accuracy, Time
Auditory 1- Back Task	Memory	90	Accuracy, Time

Visual 1- Back Task	Memory	60	Accuracy, Time
Judgement of Line Orientation Test	Visuospatial	120	Accuracy, Time
Auditory Instant Recall Task	Attention	120	Accuracy, Time
Symbol Digit Modalities Test	Attention	90	Accuracy, Time
Picture Matching Task	Attention	120	Accuracy, Time
Digital Semantic Judgment Task	Semantic Memory	60	Accuracy, Time
Auditory Delayed Recall Task	Attention	60	Accuracy, Time
Common Problem Judgment Test	Global cognitive Function	225	Accuracy, Time
Synonym Judgment Task	Semantic Comprehension	80	Accuracy, Time
Digital Association Judgment Task	Executive Function	60	Accuracy, Time

Feature extraction and selection

EEG feature extraction and selection

ApEn is a statistic that can be estimated from the discrete-time sequences to quantify the complexity or irregularity of the system. It is resistant to short strong transient interferences (outliers) such as spikes. ApEn is less sensitive to noise and can be used for short-length data. Given the embedding dimension m, the m-vector (i) is defined as

$$x(i) = [x(i), x(i+1), \ldots, x(i+\text{m-1})],\ i = 1, \ldots, \text{N-m}+1 \quad \text{(S1)}$$

Where N is the number of data points. The distance between any two of the above vectors, (i) and (j) is defined as

$$d[x(i),x(j)] = \max_{k} \left| x(i+k) - x(j+k) \right| \quad \text{(S2)}$$

where $|.|$ denotes the absolute value. Considering a threshold level of β the number of times, $M^{m}(i)$,that the above distance satisfies $d[x(i), x(j)] \le \beta$ is found. This is performed for all i For the embedding dimension m,

$$\xi^{m}_{\beta}(i) = \frac{\mathrm{M}^{m}(i)}{N-m+1} \quad for\, i = 1,\ldots, N-m+1 \quad \text{(S3)}$$

Then, the average natural logarithm of $\xi^{m}_{\beta}(i)$ is found as

$$\Psi^{\mathbf{m}}_{\beta} = \frac{1}{\mathbf{N}-\mathbf{m}+1} \sum_{\mathbf{i}=1}^{\mathbf{N}-\mathbf{m}+1} \ln \xi^{\mathbf{m}}_{\beta}(\mathbf{i}) \quad \text{(S4)}$$

By repeating the same method for an embedding dimension of $m+1$, the AE will be given

as

$$\mathbf{AE}(\mathbf{m},\beta) = \lim_{N\to\infty}(\psi_{\beta}^{\mathbf{m}} - \psi_{\beta}^{\mathbf{m}+1}) \tag{S5}$$

In practice, however, *N* is limited and therefore the AE is calculated for *N* data samples. In this case the AE depends on *m*, *β*, and *N*, i.e.

$$\mathbf{AE}(\mathbf{m},\beta,\mathbf{N}) = \psi_{\beta}^{\mathbf{m}} - \psi_{\beta}^{\mathbf{m}+1} \tag{S6}$$

The embedding dimension can be found as previously mentioned. However, the threshold value has to be set correctly. In some applications the threshold value is taken as a value between 0.1 and 0.25 times the data standard deviation.

Multiscale entropy (MSE) is an effective method to measure the complexity of time series, which can directly extract the pattern information contained in the original signal. To compute the MsEn biomarker of an N-sample EEG data sequence x (1), x (2), . . ., $x(N)$, consecutive coarse-grained time series is constructed by averaging a successively increasing number of data points in non-overlapping windows. Each element of the coarse-gained time series, $\mathbf{y}_j^{(\tau)}$, is calculated accordingly to the following equation:

$$\mathbf{y}_{\mathbf{j}}^{(\tau)} = 1/\tau \sum_{\mathbf{i}=(\mathbf{j}-1)\tau+1}^{\mathbf{j}\tau} \mathbf{x}_{\mathbf{i}} \tag{S7}$$

where τ represents the scale factor and $1 \leq \mathbf{j} \leq \mathbf{N}/\mathbf{t}$ For scale 1, the coarse-grained time series is simply the original time series. Sample entropy (SampEn)(Mahajan, Morshed, & Informatics, 2015), a refinement of the original ApEn statistics, was calculated for each coarse-grained time series plotted as a function of the of scale factor τ.

Lempel-Ziv complexity (LZC) is a nonlinear dynamic method used to detect the probability of new patterns in time series. To compute the LZC biomarker of an N-sample EEG data sequence $x(1)$, $x(2)$, . . . , $x(N)$, the EEG signal is first converted into a binary string as

$$x(i) = \begin{cases} 0 & if \quad EEG(i) < M, \\ 1 & if \quad EEG(i) \geq M, \end{cases} \tag{S8}$$

where $x(i)$ is the equivalent binary value of EEG (i), i is the index of all values in the EEG signal, and M is the median value of each EEG channel. The median value is used to manage the outliers.

The binary string is then scanned from left to right until the end to produce new substrings. A complexity counter (N) is the number of new substrings. The upper bound of (N) is used to normalize (N) to get an independent value from the sequence of length N. The upper bound of (N) is N/log2(N). (N) is then normalized by (N) as

$$C(N) = \frac{c(N)}{b(N)} \tag{S9}$$

where $C(N)$ is the normalized value of the LZC and $b(N)$ is the upper bound of the $c(N)$.
